# Performance evaluation of a health insurance in Nigeria using optimal resource use: health care providers perspectives

**DOI:** 10.1186/1472-6963-14-127

**Published:** 2014-03-14

**Authors:** Shafiu Mohammed, Aurélia Souares, Justo Lorenzo Bermejo, Rainer Sauerborn, Hengjin Dong

**Affiliations:** 1 Institute of Public Health, Medical Faculty Heidelberg, Heidelberg University, Im Neuenheimer Feld 324, 69120 Heidelberg, Germany; 2 Institute of Medical Biometry and Informatics (IMBI), University Hospital Heidelberg, Heidelberg, Germany; 3 Center for Health Policy Studies, School of Public Health, Zhejiang University School of Medicine, Hangzhou 310058, P.R. China; 4 Ahmadu Bello University, Zaria, Nigeria

**Keywords:** Health insurance, Health care providers, Optimal resource use, Nigeria, Performance

## Abstract

**Background:**

Performance measures are often neglected during the transition period of national health insurance scheme implementation in many low and middle income countries. These measurements evaluate the extent to which various aspects of the schemes meet their key objectives. This study assesses the implementation of a health insurance scheme using optimal resource use domains and examines possible factors that influence each domain, according to providers’ perspectives.

**Methods:**

A retrospective, cross-sectional survey was done between August and December 2010 in Kaduna state, and 466 health care provider personnel were interviewed. Optimal-resource-use was defined in four domains: provider payment mechanism (capitation and fee-for-service payment methods), benefit package, administrative efficiency, and active monitoring mechanism. Logistic regression analysis was used to identify provider factors that may influence each domain.

**Results:**

In the provider payment mechanism domain, capitation payment method (95%) performed better than fee-for-service payment method (62%). Benefit package domain performed strongly (97%), while active monitoring mechanism performed weakly (37%). In the administrative efficiency domain, both promptness of referral system (80%) and prompt arrival of funds (93%) performed well. At the individual level, providers with fewer enrolees encountered difficulties with reimbursement. Other factors significantly influenced each of the optimal-resource-use domains.

**Conclusions:**

Fee-for-service payment method and claims review, in the provider payment and active monitoring mechanisms, respectively, performed weakly according to the providers’ (at individual-level) perspectives. A short-fall on the supply-side of health insurance could lead to a direct or indirect adverse effect on the demand-side of the scheme. Capitation payment per enrolees should be revised to conform to economic circumstances. Performance indicators and providers’ characteristics and experiences associated with resource use can assist policy makers to monitor and evaluate health insurance implementation.

## Background

National health insurance schemes (NHIS) are implemented as part of health reform and as strategies aimed towards providing effective and efficient health care for citizens in many low and middle income countries (LMICs). Some LMICs are still in the early stages of implementation, while others are in the middle and late stages. Despite challenges in implementation, most LMICs such as Nigeria, India, and Kenya have increased government spending as a percentage of total health expenditure (THE) between 1 and 3 percentage points [1]. Ghana, Rwanda, and Indonesia have increased THE between 5 and 10 percentage points after launching reforms [1]. The incremental approach to risk pooling, used by most LMICs, commences with multiple pools for different target populations [1].

In Nigeria, health insurance was introduced to the citizens in mid-2005. Presently, revenue for implementation of the NHIS relies on formal-sector payroll contributions and general government revenues, despite the challenges of tax collection [1]. The formal-sector programme is paid for with funds created by pooling the contributions of employees and employers [2]. Contributions to the program are 15% of the employee’s basic salary, with the employee contributing 5% and employers contributing the remaining 10% [2-4]. Nigeria has adopted the incremental approach of risk pooling, using multiple stages which would later incorporate other population groups in different NHIS programs [1]. Purchasing services, in the NHIS, is accomplished through a mix of public and private providers who are reimbursed by purchasing-agency resources controlled by health management organizations (Hmos).

There have been virtually no in-depth studies in low, middle and high income countries that deal with the performance evaluation of health insurance using optimal-resource-use (ORU) domains. The performance of health insurance schemes, which begins to dominate health care financing in LMICs, demands greater attention in the contemporary context. Fundamental and structural problems associated with ORU domains need to be understood, and effective solutions proffered. LMICs would benefit from common, comparable standards for measuring performance of schemes related to ORU, either across countries or across regions within a country, which could guide midcourse policy corrections and improve implementation [1].

NHIS performance measures and their indicators are instrumental in assisting policy and decision makers in identifying target groups, clarifying objectives, defining measures of performance, and developing performance information systems [5]. These performance measures include revenue collection, pooling and purchasing in terms of ORU [1,6-11]. Presently, there is a lack of evidence in LMICs to evaluate the performance of health insurance using optimal-resource-use as an outcome measure. Furthermore, little is known on how to evaluate this optimal-resource-use.

ORU is a health financing target used to evaluate how well social health insurance performs [6,7]. The WHO has proposed the model of optimal-resource-use domains to monitor and evaluate the performance of health insurance schemes during the transition period of implementation [6-11], but evidence from in-depth investigations in LMICs is still lacking. These ORU domains encompass administrative efficiency, active monitoring mechanism, provider payment mechanisms, and benefit package inclusions [6-11]. ORU domains of health insurance schemes accentuate the importance of monitoring and evaluation, but the appraisals are usually not known, and the suitability and practicality have not yet been explored and examined. Moreover, the supply-side of a health insurance system and its influencing factors and characteristics are mostly neglected. Implementers, policy and decision makers should understand the key characteristics and potential factors influencing the insurance supply-side to improve the schemes’ implementation.

This paper examines the performance of Nigeria’s NHIS related to ORU domains based on providers’ experiences. Moreover, the paper addresses the key characteristics and potential issues challenging the Nigeria’s NHIS implementation according to the providers’ perspectives. As this study is exploratory in nature, we hypothesized that the ORU domains ratings would be influenced by certain provider personnel experiences and associated characteristics at individual level. These hypotheses and related assumptions are supported by the theoretical framework of performance indicators for the implementation of social health insurance and related literatures [3,4,6,7,12,13]. This study was carried out to evaluate the progress of implementation of the health insurance scheme related to ORU domains.

## Methods

### Study setting

This retrospective cross-sectional study was conducted in Kaduna state between August and December 2010. The state is located in the central region of Nigeria, which borders the federal capital territory (Abuja). Kaduna state was chosen because it has one of the highest numbers of providers’ health facilities (N = 116) out of the country’s total number (N = 2646) accredited to provide both primary and secondary healthcare in the NHIS at the time of the study, as identified by the NHIS regulatory agency. Most of the health facilities in the state have been accredited since the inception of the NHIS in Nigeria.

### Sampling

A two-stage sampling procedure was used to select the study participants. We employed simple random sampling at each stage to reduce selection bias. The first stage involved selecting only 68 health facilities that were accredited by the NHIS in the three years preceding the survey (NHIS 2010 regional office data, Kaduna). The second stage involved the random selection of 509 provider personnel that were actively involved in health insurance activities for at least three years in the randomly selected health facilities. This sampling was proportionally distributed across the selected health facilities based on number of personnel working within the health facility. Verification of the health facilities was carried out in collaboration with the NHIS regional officers in Kaduna and NHIS headquarters in Abuja-Nigeria. Four hundred and sixty-six provider personnel from 57 accredited health facilities were interviewed, and this was done to ensure facility-size representation at individual level.

### Questionnaire

A pre-tested interviewer-administered questionnaire was used. The questionnaire consisted of two sections: (i) personal characteristics of each provider (at individual level) related to the optimal-resource-use of the insurance scheme and (ii) information on the evaluated outcome of health insurance performance including types of optimal-resource-use domains as rated by the respondents. This structured questionnaire was designed to particularly address the study objectives. The questionnaire was pilot tested on ten respondents from 10 health facilities. Furthermore, the questionnaire was field-tested five times prior to this study. Following pilot-testing, minor modifications were made to the questionnaire, including revisions to the sequence and phrasing of some questions to make them clearer to understand. The detailed explanation of the variables and their measurements used in the questionnaire are presented below. In order to enhance clarity and limit misinterpretation of terms by the respondents, questionnaire manuals, defining various key terms and optimal-resource-use domains, were incorporated as addenda for the participants (see Table 1). The questionnaire was administered to the providers at individual level. To provide a more constructive explanation of outcome and explanatory variables, we caution that the use of indicators, to judge performance of insurance schemes related to ORU, requires a clear understanding of the organizational structure, operational arrangements of the system, suitability for purpose and context, and caveats about interpretation. In this study, the variables were primarily generated according to the insurance scheme in Nigeria contextually. The literature identifies an unmet need for a rigorous means of selecting indicators according to suitability for purpose and use contextually [14].

**Table 1:** Synopsis of definitions related to “optimal-resource-use” domains by the providers

**Domain name**	**Variable used**	**Definition (provided in the interview manual)**
**Administrative efficiency**	Prompt arrival of insurance funds	Regular monthly reimbursement of capitation payment made by the Hmos^*^ to the health facilities or hospitals, and fee-for-service payment in case of referral.
	Promptness of the referral system	Efficient and rapid (24 hours) response of the Hmos^*^ to the health care provider, when patients are to be referred for secondary or tertiary care in the insurance scheme.
Active monitoring mechanism	Claims review (as a proxy)	Monthly cross-checking that enrolees received all necessary treatments and diagnostic tests covered by the health insurance and all prescribed drugs that are included in the NHIS^*^ drug list.
**Provider payment mechanism**	Capitation payment method	Total monthly amount of money (fixed per enrolee) received by the health provider or hospital from the Hmos^*^. This total fixed sum excludes payments for referrals and 10% of total cost of drugs paid already by the patient during hospital visit.
	Fee-for-service payment method	This is termed an amount of money reimbursed by the Hmos to the health provider facility or hospital per episode of specialty, secondary or tertiary care administered to the enrolee. This amount of money excludes payments for capitation and 10% of total cost of drugs paid already by the patient during hospital visit.
Benefit package	Benefit package inclusion	Includes all treatments and diagnostic tests covered by the health insurance plus all drugs prescribed by doctors or nurses as included in the NHIS^*^ drug list. This benefit package includes out-patient care, in-patient care, specialized care (eye and dental care), and prescribed medications and consumables. However there are exclusions such as dialysis, cancer treatment, mental disorders, branded medications and ostentatious consumables (as specified by the regulatory agency).


*Abbreviations:Hmos health management organizations, *NHIS* national health insurance scheme.

Sources: NHIS [2]; NHIS [3,4]; Carrin and James [6-8].

### Explanatory variables

In the questionnaire, respondents were asked, “Which professional category of health provider relating to assigned duties and responsibilities do you belong to?” including doctor, nurse, pharmacist, record officer, and other administrative staff [4,13]. The responses on type of facility and number of enrolees [4,13], were obtained from both the providers and NHIS official records (see Table 2). Respondents were asked how often they interact or have contact with enrolees seeking health care [13], including the last 1 month, and also the last 12 months. Responses to each of these items were categorized “Weekly” and “Every two weeks” which were included in the questionnaire. This interaction indicates the extent to which the respondent is connected to the enrolees and exposure to resource use activities [4,13].

**Table 2:** Characteristics and experiences of HCPs^* ^**to investigate health insurance optimal-resource-use activities**

**Independent variables**	**Categories**	**Number**	**Percentage**
Total cases		466	100%
Providers profession^1^	Medical	293	63%
	Non-medical	173	37%
Type of facility	Public	281	60%
	Private	185	40%
Number of enrolees in providers^*^ facility	1000 or less	63	14%
	More than 1000	403	86%
Last 1 month contact with enrolees by providers	Weekly	389	85%
	Every two weeks	77	15%
Last 12 months contact with enrolees by providers	Weekly	395	83%
	Every two weeks	71	17%
Improvement in waiting times of enrolees	Yes	370	79%
	No	96	21%
Providers received enrolees^*^ list quarterly^2^	Yes	324	70%
	No	142	30%
Facility inspection in the last 12 months^3^	≤2 times	377	81%
	>2 times	89	19%
Overall annual inspection^3^	Yes	142	30%
	No	324	70%
Providers 12 months contact with referral	Monthly	213	46%
	≥2 months	253	54%
12 months availability of insurance funds	Monthly	366	79%
	Every four months	100	21%
3 years availability of insurance funds	Monthly	370	79%
	Every four months	96	21%
Information on full entitlement made available by NHIS^*^	Agree	119	26%
	Disagree	347	74%
Health insurance to revise capitation payment^4^	Agreed	435	93%
	Disagree	31	7%
Overall capitation payment amounts ratings	Good	339	73%
	Bad	127	27%

*Abbreviations:NHIS national health insurance scheme regulatory agency.

^1^Profession means the assigned duties and responsibilities of providers. Again, non-medical is administration personnel including record officers with no medical responsibility, but involved in resource use activities.

^2^Quarterly means every three months of a year instalmentally.

^3^Inspection means quality assurance checks of providers’ activities conducted by external monitors.

^4^Providers opinion on capitation payment per enrolee to be revised by the NHIS regulatory agency.

To investigate the providers’ previous experiences, respondents were asked whether there were improved waiting times to see the doctor or nurse (as perceived by the providers) [4,13], receive enrollee lists quarterly and overall annual inspection of their activities. The responses to each of these items were dichotomized “Yes” and “No” which was included in the questionnaire. Relating to frequency of inspection [3,4], respondents were further asked how many times they were inspected by the health insurance regulatory monitors in the last 12 months. Responses to this item ranged between “none”, “1 to 2 times”, “3 to 4 times”, “5 to 6 times”, and “At least 7 times”. However, in our analysis, this response was converted into 2 categories of “At most 2 times” and “More than 2 times”. This frequency indicates the extent to which the respondent is exposed to the inspection activities of the insurance scheme [4,13]. Concerning referral, respondents were asked how often they interact or deal with activities of referring enrolees/patients for secondary or tertiary care [12]. Responses to this item ranged between “Monthly”, “Every 2 months”, “Every 3 months”, “Every 4 months”, and “At least every 5 months”. Again, in our analysis, this response was converted into 2 categories of “Monthly” and “At least every 2 months”. Regarding availability of funds, respondents were asked how often insurance funds are made available, including the last 12 months, and also the last 3 years. The responses to each of these items were dichotomized “Monthly” and “Every 4 months”. There is an inconsistent variation of insurance funding available to the providers [3,4]. Respondents were asked their concerns and opinions about the availability of information on full entitlements, their desire for the health insurance scheme to review capitation payments to ensure good quality service, and the overall ratings of capitation payment amounts [4,13].

### Outcome variables

In the questionnaire, the outcome variables (see Table 3) include providers’ rated measures of ORU domains. Our study is supporting the theoretical framework of performance indicators for NHIS implementation [6,7]. Table 1 presents a synopsis of definitions related to optimal-resource-use domains (also provided in the interview manual) used during the study. All questions that were asked on ORU domains are related to the definitions in Table 1. Administrative efficiency was determined by prompt arrival of insurance funds and promptness of the referral system [2-4,6,7]. Respondents were asked, “Overall, how would you rate the prompt arrival of insurance funds?”, and “Overall, how would you rate the promptness of referral system within the insurance scheme?”. Active monitoring mechanism was derived using review of claims (as a proxy) [2-4,6,7]. The respondents were asked, “Overall, how would you rate claims review done by the regulatory monitors in the last 12 months?” Provider payment mechanism was characterized as capitation and fee-for-service (in the case of referrals) payment methods [2-4,6,7]. Here, respondents were asked, “In general, how would you rate the capitation payment method during the last 12 months?”, and “In general, how would you rate the fee-for-service payment method for referral during the last 12 months?” Benefit package inclusion was defined as all treatment and diagnostic tests covered by the health insurance [2-4,6,7]. Again, the respondents were asked, “Overall, how would you rate all the treatment and tests covered by the insurance in form of benefit package?” All the responses to each of these items ranged between “very good”, “good”, “moderate”, “bad”, and “very bad”.

**Table 3:** Types of optimal-resource-use domains* as rated by the providers’ respondents (n = 466)

**Domains**	**Variables**	**Provider’s ratings; N (%)**			
		**Bad**	**Percent**	**Good**	**Percent**
Administrative efficiency	Promptness of referral system	92	20%	374	80%
	Prompt arrival of insurance funds	33	7%	433	93%
Active monitoring mechanism	Claims review	293	63%	173	37%
Provider payment mechanism	Capitation payment method	22	5%	444	95%
	Fee-for-service payment method	177	38%	289	62%
Benefit package	Benefit package inclusions	14	3%	452	97%

^*^The definitions and measurements of optimal-resource-use domains and variables by the providers are presented in Table 1 and the method section.

### Measures and data analysis

The variables of ORU domains were measured using Likert-type items of five ordered categories, rated from one (very good) to five (very bad). These ordered categories were transformed, summated and the responses converted into 2 categories of one (good) and two (Bad), as presented in Table 3. We employed the “observational model” and generated the cut-off threshold based on distribution of responses [15-21]. The McNemar and marginal homogeneity tests that were used emphasized the comparison of the distributions [15,16,18,22]. The categories of “very good” and “good” were termed as one (good), while the “moderate”, “bad”, and “very bad” were termed as two (bad). This conversion was carried out because the resultant binary variables reflected a meaningful distinction in practical terms [15-21].

The list of explanatory variables is shown in Table 2. The explanatory variables were selected according to the study hypotheses. We tested for collinearities and eliminated only one variable with a correlation above 0.75. The eliminated variable was “Last 1 month contact with enrolees by providers”. It strongly correlated with “Last 12 months contact with enrolees by providers”. We kept the variable “Last 12 months contact with enrolees by providers” because of its relevance to the study since health insurance aimed to promote utilization of health services. Moreover, this variable would provide better additional information on the providers’ experiences with the insurance scheme. We analysed each of the explanatory and ORU domain variable as a dichotomous category. Logistic regression was used to explore the influential factors associated with the ORU. Stepwise selection using backward procedure was applied to identify the associations in the multiple logistic regression. Our reasons for this approach were that, (i) this is the first in-depth research study that investigated ORU domains of the NHIS based on providers experiences within LMICs context practically, (ii) we are investigating and quantifying the most relevant perceived providers’ (individual level) factors which are potentially associated with the resource use activities, and (iii) that these results should be empirically interpreted. Furthermore, the final decision on which indicators were candidates for removal from the model was largely determined by the context. We reasonably considered the relevant model specifications and assumptions at various stages of the statistical analyses [23,24]. Associations between the outcome and explanatory variables were assessed using odds ratios, 95% confidence intervals and probability values. A p-value of 0.05 was used as the threshold for statistical significance. The list of independent variables is shown in Table 2, and outcomes in Table 3. The STATA program (STATA® 12.1, 2011; StataCorp LP., 4905 Lakeway Drive, College Station, Texas, 77845 USA) was used to carry out the analyses.

This study protocol was approved by the Ethics Commission of Heidelberg University-Germany [S-035/2009], University Research Ethics Committee ABU-Nigeria [VC/P. 18890] and the NHIS Headquarters Abuja-Nigeria [NHIS-467].

## Results

### Providers’ characteristics and experiences related to health insurance resource use activities

Interviewees and their facilities needed to be accredited as providers and actively involved in health insurance activities of the NHIS for at least three years. Among the 509 providers’ personnel from the 68 health facilities requested to participate, 466 providers from 57 health facilities responded. Here, at the first stage sampling of accredited health facilities, we achieved 83.8% response rate, while at the second stage of sampling provider personnel we achieved 91.6% response rate.

Of the 466 providers’ personnel included in this study, 60% were public providers and 40% were private providers, presented in Table 2. Most of the providers were from health facilities with at least 1000 enrolees registered in the scheme. Providers’ personnel mostly had contact with enrolees in the last month preceding the survey. Most of the providers reported that waiting times for outpatient care had improved. By contrast, only 30% of the providers reported that overall inspection was conducted annually. The majority of providers reported that insurance funds were mostly available during the last 12 months and 3 years, respectively. This was done to assess consistency and disparity during the period. A reasonable minority of the providers (26%) reported that information on full entitlement was provided. Most of the providers (93%) agreed that capitation payment per enrolee should be revised by the NHIS regulatory agency.

### Performance of optimal-resource-use domains evaluated by providers according to their perspectives

The evaluated optimal-resource-use domains and provider responses are presented in Table 3. In the administrative efficiency domain, both “promptness of the referral system” and “prompt arrival of insurance funds” were rated “good”, 80% and 93%, respectively, by the providers. The active monitoring mechanism (claims review) was considered “bad” (63%) by the providers. In the provider payment mechanisms, capitation payment method was rated highly (95%), but the fee-for-service payment method was only rated “good” at 62 percent. The benefit package inclusion was rated nearly unanimously as “good” (97%).

### Providers’ characteristics and experiences associated with the insurance optimal-resource-use domains

The results in this section were obtained using multiple logistic regression models and are presented in Table 4. Respondents’ characteristics and functions associated with the insurance optimal-resource-use domains are depicted in relation to the NHIS implementation strategies. In the administrative efficiency domain, providers with lower numbers of enrolees were less likely to report prompt arrival of insurance funds (reimbursement) to their facilities (OR = 0.28, 95% CI 0.11 – 0.68). However, providers with 12 months and 3 years funds availability were more likely to have better reimbursement to their health facilities (OR = 3.50, 95% CI 1.46 – 8.36 and OR = 2.43, 95% CI 1.02 – 5.89, respectively). Regarding promptness of the referral system, the most important associated factor was overall annual inspection. Providers that underwent annual inspections of their health facilities were more likely to report better promptness of the referral system in the NHIS than those with no annual inspection (P <0.0001, OR = 4.42, 95% CI 2.02 – 9.68).

**Table 4:** Multiple logistic regression analyses of providers characteristics and experiences (at individual level) in relation to optimal-resource-use domains

	**Administrative efficiency**						**Active monitoring mechanisms**			**Provider payment mechanisms**						**Benefit package**		
	**Prompt arrival of insurance funds**			**Promptness of referral system**			**Claims review**			**Capitation payment**			**Fee-for-service payment**			**Benefit package inclusions**		
	**P-value OR**	**(95% CI)**		**P-value OR**	**(95% CI)**		**P-value OR**	**(95% CI)**		**P-value OR**	**(95% CI)**		**P-value OR**	**(95% CI)**		**P-value OR**	**(95% CI)**	
Providers profession				0.05														
Non-medical				Ref.														
Medical				1.67	1.02	2.78												
Number of enrolees in providers facility	0.005						0.01											
>1000	Ref						Ref											
≤1000	0.28	0.11	0.68				2.40	1.20	4.80									
12-month contacts with enrolees													0.014			0.008		
Every 2 weeks													Ref.			Ref.		
Weekly													0.46	0.25	0.85	4.48	1.47	13.68
Improved waiting times of enrolees	0.03			0.03												0.002		
No	Ref.			Ref.												Ref.		
Yes	2.47	1.07	5.69	1.81	1.05	3.11										5.53	1.84	16.59
Receive enrolees list quarterly							0.05											
No							Ref.											
Yes							0.55	0.32	0.98									
Overall annual inspection				<0.0001									<0.0001					
No				Ref.									Ref.					
Yes				4.42	2.02	9.68							4.37	2.57	7.43			
12-month contact with referral				0.005														
≥ 2 months				Ref.														
Monthly				2.13	1.25	3.63												
12-month funds available	0.005																	
Every 4 months	Ref.																	
Monthly	3.50	1.46	8.36															
3-year funds available	0.05									0.031								
Every 4 months	Ref.									Ref.								
Monthly	2.43	1.02	5.89							2.71	1.10	6.68						
Information on entitlement available							<0.0001						<0.0001					
Disagree							Ref.						Ref.					
Agree							82.99	37.46	183.85				4.63	2.56	8.36			
NHIS to revise capitation payment							<0.0001						0.004					
Disagree							Ref.						Ref.					
Agree							0.15	0.06	0.35				0.16	0.05	0.57			
Providers ratings of capitation amounts	0.002			0.002						<0.0001								
Bad	Ref.			Ref.						Ref.								
Good	3.40	1.55	7.47	2.22	1.34	3.69				7.13	2.70	18.86						
n	466			466			466			466			466			466		
R^2^	0.213			0.118			0.424			0.142			0.161			0.123		
Model test: LR χ^2^	50.74; p < 0.001			54.55; p < 0.001			260.41; p < 0.001			25.23; p < 0.001			99.53; p < 0.001			15.46; p < 0.001		
log likelihood	−93.81			−204.24			−177.17			−76.024			−259.65			−55.13		

The ranking of response variables based on their strength of association with the active monitoring mechanism, in descending order was: “information on full entitlement provided by NHIS” (P <0.0001), “capitation payment per enrolee to be revised by the NHIS” (P <0.0001), “number of enrolees in the providers facility” (P = 0.01), and “providers received enrolees list quarterly” (P = 0.05). Providers with fewer enrolees, and those that received the information on full entitlement which was provided by the NHIS, were more likely to report better claims review. However, providers who regularly received enrolee lists, and were of the opinion to revise the capitation payment per enrolee, were less likely to report better insurance claims.

The provider payment mechanism was examined by capitation and fee-for-service (in case of referral) payment methods. Providers with three years fund availability in their facility (OR = 2.71, 95% CI 1.10 – 6.68) and those with good ratings of their capitation payment amounts (OR = 7.13, 95% CI 2.70 – 18.86) were more likely to report better capitation payment method. The ranking of response variables based on their strength of association with the fee-for-service payment method, in descending order was: “information on full entitlement provided by the NHIS” (P <0.0001), “overall inspection of providers activities was conducted annually” (P <0.0001), “capitation payment per enrolee to be revised by the NHIS” (P <0.004), and “providers twelve months contact with the enrolees” (P <0.014). Providers with regular annual inspection of their health facilities and those that indicated the information on full entitlement was provided by the NHIS were more likely to report better fee-for-service payment method in the insurance scheme. By contrast, providers’ who regularly had contact with the enrolees, and those advocating for a revision of the capitation payment per enrolee, were more likely to report poor fee-for-service payment procedures in the NHIS.

Providers’ twelve months contact with the enrolees was positively associated with the benefit package domain (OR = 4.48, 95% CI 1.47 – 13.68). Providers who regularly had contact with the enrolees were more likely to report better benefit package inclusion in the insurance scheme.

## Discussion

Considering the supply-side of a health insurance system has direct and indirect effects on the demand-side of the system, evaluating performance of the NHIS using ORU according to providers’ perspectives is relevant during implementation, and could stimulate the need for reform. This study aimed to assess the performance of the health insurance program using optimal-resource-use (ORU) based on providers’ perspectives. We demonstrated that ORU domains were useful in evaluating the performance of health insurance schemes during implementation. Our study corroborates the importance of these ORU domains and provides support to the theoretical framework of performance indicators for the implementation of national health insurance schemes in LMICs [6,7]. To our knowledge, this is the first practical in-depth study to approach the investigation of ORU domains based on providers’ perspectives, and methodological points of view in the context of NHIS performance evaluation in LMICs. Considering the exploratory nature of this study, we further examined providers’ characteristics and concerns related to ORU of the insurance scheme based on their experiences.

Stakeholders on the supply-side of the insurance system, particularly providers and Hmos (insurers), are the most relevant and specific target-groups to evaluate the performance of health insurance related to ORU. We identified weaknesses of the fee-for-service payment method and of claims review in the provider payment and active monitoring mechanisms, respectively. The NHIS regulatory agency should consider appropriate reform measures to deal with these shortcomings in the scheme. In 2005, Carrin and James argued that the most important key factors affecting the transition of NHIS implementation is the system’s ability to adequately administer and manage the insurance [8].

### Administrative efficiency

Administrative efficiency was investigated in our study using two proxies, including prompt arrival of insurance funds and promptness of the referral system in the NHIS. The most interesting finding was that promptness of the referral system performed poorer than prompt arrival of insurance funds to providers in the insurance Scheme. A possible explanation for this might be delaying administrative procedures and payments for referrals in the NHIS. Recent studies have explained that late reimbursement of insurance funds to providers results in administrative inefficiency, which is dependent on administrative expenditure [25]. By promoting maximum cost recovery, the providers are motivated to function efficiently to remain within their predetermined budgets. In addition, these funds might be utilized more effectively to provide quality services to the clients. Nevertheless, it would be inappropriate to equate the observed optimal-resource-use with low administrative efficiency and delayed referral in the system [7,25]. Simple and affordable administrative protocols might be used to restructure the referral system procedures by the regulatory agency.

Providers with fewer enrolees encountered difficulties with reimbursement which caused poor administrative efficiency, according to our findings. In this study, availability of insurance funds and capitation amounts acquired by the providers might have augmented the providers rating of prompt arrival of insurance funds. This result may be explained in that providers with better availability of insurance funds and also acquired capitation amounts in their facilities were likely to be promptly reimbursed in the insurance scheme.

In our study, the NHIS referral system was found to be less administratively efficient than the insurance reimbursement, according to the providers. The results indicate that the higher the scores are for improved waiting times and inspection of the providers, the better the promptness of the referral system. These findings suggest that regular inspection of providers might possibly assist in preventing delays in referrals of the NHIS.

### Active monitoring mechanism

The active monitoring mechanism was poorly rated by the providers, according to our findings. A possible explanation for this might be that monitoring mechanisms such as patient appeals, peer-review committees and active claim reviews were not fully established in the NHIS. The presence of a claims review process ensures that insurance claims are independently reviewed by appropriate personnel and that claims made by healthcare providers are justified [7,26]. The NHIS should adopt peer-review committees, not fully planned in the scheme, and reactivate the claims reviews that might help improve the active monitoring mechanism during implementation.

This study found the fewer the number of enrolees with the providers, the better the claims review. However, a possible explanation could be that providers who are overcrowded with enrolees tend to have difficulties with the claims review, which might in turn lead to negative effects on the monitoring mechanism of the insurance scheme. This study demonstrates that better availability of information on full entitlement, leads to a better active monitoring mechanism in the insurance scheme. The findings also revealed that negative consequences due to poor capitation payment resulted in a negative effect on the claims review. In 2000, Mills et al. cautioned the drawback of capitation payment is that insurance controls costs by transferring the risk to the health care providers [13].

### Provider payment mechanism

Capitation payment is the most common method that is used to pay providers in the NHIS of Nigeria. A most interesting finding was that providers supported the capitation payment method more than fee-for-service (FFS) payment method. Although each payment mechanism has its relative strengths and weaknesses, revising these methods may encourage the providers to provide appropriate healthcare to their patients [7]. Conceptually, the capitation method is expected to encourage underproduction and the FFS method to promote overproduction of health care services in insurance schemes. Studies have suggested that insurance schemes could achieve better performance through integrated referral systems with effective monitoring [7]. Another important finding was that providers who received insurance funds regularly, favored the capitation payment method. This observation provides further evidence that the capitation method likely encourages providers’ underproduction of healthcare services in the NHIS [7]. Moreover, distortion of government-set prices which are lower than the real costs, gives lower incentives for providers that may result in under-utilization of health services by clients [27].

The FFS payment method is applied to the referral system of the NHIS in Nigeria, because patients’ referrals to secondary or tertiary levels of care are paid by the FFS method. Our findings revealed that providers who had relatively more contact with the enrolees rated the FFS method poorer than those who had less contact with the enrolees. This is evidence that providers are either displeased or encountered obstacle with the FFS method during patients’ referrals. A further possible explanation might be that the FFS method discouraged providers to further refer patients in the insurance scheme. This may suggest that regular inspection in the NHIS might facilitate appreciation of the FFS method by identifying and resolving providers’ impediments. Moreover, if the capitation payment is revised and the FFS method restructured by the NHIS regulatory agency, then the providers’ motivation and patients’ utilization of health care services might improve in the insurance scheme.

### Benefit package inclusion

The benefit package inclusion was rated highest among all ORU domains by the providers, according to our findings. Recent studies have explained that better performance of insurance schemes is associated with evidence that the benefit package is comprehensive and in accordance with society’s preferences, so that resources are best utilized [7]. We found that regular contact of providers with enrolees positively influenced their rating of the NHIS benefit package. Several studies have revealed that better provider-client interactions have an effect on the benefit package and the availability of the full benefit package improves these interactions [5-8,28]. However, some studies have suggested that the benefit package could be incrementally developed according to the scheme’s ability to pay [28], changing needs, values and economic circumstances [5-8]. Countries such as South Korea, Singapore and Thailand have experienced the incremental development of benefit packages [28] to improve performance of their insurance schemes. Moreover, this approach has been supported by the World Health Organization and the World Bank [29-31].

The results of this study should be interpreted considering its limitations. First, the performance evaluation of health insurance using ORU, according to providers’ perspectives, might not comprehensively conform to societal perspectives. However, problems on the supply-side of the insurance scheme have the tendency to negatively affect the demand-side, leading to unintended consequences. Second, because of its cross-sectional design, the associations identified here do not imply causality. Third, the possible dependence of responses by facility was a limitation of the present study. However, when the clustering effect was taken into account in the logistic regression models, the estimates were practically identical. For instance, the estimated OR for prompt arrival of insurance funds by ratings of capitation amounts changed from 3.40 (95% CI 1.55 – 7.47) to 3.40 (95% CI 1.62 – 7.13). For the sake of simplicity and the perceptions of individuals, estimates were reported without considering this clustering effect. Future research may explore opportunities to study ORU with more focus on the facility level, to account for the multi-level nature of the system. Further similar studies should be conducted across Nigeria and other LMICs to explore regional differences and variations related to NHIS periodic performance.

## Conclusions

Fee-for-service payment method and claims review, in the provider payment and active monitoring mechanisms, respectively, performed weakly according to the providers’ (at individual-level) perspectives. This might not comprehensively conform to societal perspectives. However, problems on the supply-side of the insurance scheme have the tendency to negatively affect the demand-side, leading to unintended consequences. Among several providers’ characteristics and experiences which significantly influenced ORU, it seems relevant that providers with fewer enrolees encountered particular difficulties with reimbursement. The capitation payment per enrolee should be revised to conform to economic circumstances. Periodic identification of providers’ characteristics and experiences that influence ORU can assist in guiding policy and implementation of reforms.

## Competing interests

The authors declare that they have no competing interests.

## Authors’ contributions

SM, HD, AS, JLB and RS conceptualized and designed the study protocols. SM and HD carried out the field study work. SM, HD and JLB analysed and interpreted the data. SM drafted the manuscript. The findings were critically reviewed by AS, HD, JLB and RS. All authors read and approved the final manuscript.

## Pre-publication history

The pre-publication history for this paper can be accessed here:

http://www.biomedcentral.com/1472-6963/14/127/prepub
